# Quantification of the pulmonary vascular obstruction index on ventilation/perfusion lung scintigraphy: Comparison of a segmental visual scoring to the Meyer score

**DOI:** 10.3389/fmed.2022.970808

**Published:** 2022-10-26

**Authors:** Romain Le Pennec, Cécile Tromeur, Charles Orione, Philippe Robin, Raphaël Le Mao, Christophe Gut-Gobert, Grégoire Le Gal, Pierre Yves Salaün, Pierre Yves Le Roux

**Affiliations:** ^1^U1304 (GETBO) IFR 148, CHRU de Brest, Service de Médecine Nucléaire, Université de Bretagne Occidentale, Brest, France; ^2^Département de Médecine Interne et Pneumologie, U1304 (GETBO), CHRU de Brest, Université de Bretagne Occidentale, Brest, France; ^3^Centre d’Investigation Clinique, Centre Hospitalier Régional et Universitaire de Brest, Brest, France; ^4^Department of Medicine, Ottawa Hospital Research Institute, University of Ottawa, Ottawa, ON, Canada

**Keywords:** V/Q—ventilation/perfusion, lung scan ventilation/perfusion, pulmonary vascular obstruction, quantification, lung scintigraphy

## Abstract

**Introduction:**

Quantifying the pulmonary vascular obstruction index (PVOI) is essential for the management of patients with pulmonary embolism or chronic thromboembolic pulmonary hypertension (CTEPH). The reference method for quantifying the PVOI with planar lung ventilation/perfusion (V/Q) scintigraphy is the Meyer score, which was validated using pulmonary angiography as a reference standard. However, it is complex to use in daily practice. In contrast, a rapid and fast quantification method consists in estimating the PVOI based on the number of segmental perfusion defects. However, the accuracy of this method has never been evaluated. In this study, we aimed to compare PVOI quantification on planar V/Q scintigraphy assessed by a segmental visual scoring (SVS) to the Meyer score.

**Materials and methods:**

The eligible study population consisted of consecutive patients who underwent planar V/Q scan for CTEPH screening. A central review was performed by three nuclear medicine physicians. PVOI was assessed by summing the number of segmental perfusion defects or equivalent (2 sub-segments = 1 segment = 5%) and by Meyer’s method. The two interpretations were performed 6 months apart. A Spearman rank correlation coefficient was calculated to evaluate correlation between the two measurement methods. An intra-class correlation (ICC) was calculated to assess agreement. A Bland et Altman plot analysis was used to evaluate agreement between the two measurements.

**Results:**

A total of 226 V/Q scans were interpreted. Spearman rank correlation coefficient between SVS and Meyer was 0.963 (95%CI 0.952–0.971) for mismatched perfusion defects and 0.963 (95%CI 0.953–0.972) for perfusion defects regardless of ventilation. Intra-class correlation (ICC) for agreement was 0.978 (95%CI 0.972–0.983) for mismatched perfusion defects and 0.968 (95%CI 0.959–0.976) for perfusion defects regardless of ventilation. In Bland & Altmann analysis, the mean difference between the SVS method and the Meyer score was 0.42 and 0.61 for the mismatched or matched evaluation, respectively.

**Conclusion:**

Our study shows a high correlation, and low differences in PVOI quantification when using a segmental visual scoring (SVS) as compared to the Meyer score. The SVS has the great advantage to be easy and rapid to apply in daily practice.

## Introduction

Lung ventilation/perfusion (V/Q) scintigraphy is a well-established test for the diagnosis of acute pulmonary embolism (PE) (1, 2). It is also the imaging modality of choice to exclude chronic thromboembolic pulmonary hypertension (CTEPH) at an early stage of the algorithm for diagnosing PH (1, 3). In both clinical scenarios, the principle of interpretation of the V/Q scan is similar, based on the recognition of mismatched perfusion defects.

In patients with PE or CTEPH, there is a growing interest in quantifying the pulmonary vascular obstruction index (PVOI), i.e., the percentage of the whole lung volume with perfusion defects. Indeed, a high pulmonary vascular obstruction index (PVOI) measured at the time of PE diagnosis was associated with an increased risk of residual obstruction (4, 5) and recurrent venous thromboembolism (VTE) (6, 7). Similarly, studies suggest that residual PVOI, when measured with lung scan after a minimum of 3 months of anticoagulant therapy, might be associated with a 2- to 3-fold increased risk of recurrent VTE (4, 8, 9). In CTEPH patient, PVOI assessment at screening might also be useful as it would make it possible to follow the evolution of the obstruction, especially in patients whose clinical or hemodynamic parameters deteriorate over time. In addition, it could be a prognostic factor for post-treatment outcomes after surgery (10) or balloon pulmonary angioplasty.

The reference method for quantifying the PVOI with planar lung V/Q scintigraphy is the Meyer score, which was validated using pulmonary angiography as a reference standard (11). Each lobe is assigned a weight based on the regional distribution of pulmonary blood flow. Perfusion within each lobe is estimated by a semi-quantitative perfusion score based on the size and severity of the perfusion defect. Each lobar perfusion score is then calculated by multiplying the weight of the lobe by the perfusion score and the overall perfusion score is determined. Although being used as the reference method for PVOI quantification on planar lung V/Q scintigraphy in many studies, the Meyer score is complex to use in daily practice, time consuming and involves a certain amount of subjectivity. In particular, a semi-quantitative perfusion score from 0 to 1 (0, 0.25, 0.5, 0.75, or 1) has to be assigned for each lobe based on the size and severity of the perfusion defect, but a clear definition of how to quote this perfusion score is not provided, which may lead to interobserver variability. Thus, in a study published by Wan et al. (12), 65 participants were randomly selected for independent assessment of PVOI by a second nuclear medicine physician. The kappa coefficient for interobserver agreement between the two nuclear medicine physicians was only 0.71. As a consequence, the Meyer score is almost never used in routine clinical practice and even unknown from most practicing nuclear medicine physicians.

In contrast, another method based on a segmental visual scoring (SVS) is commonly used by nuclear medicine physicians. In this method, the score is obtained by adding 5% for each segmental perfusion defects or equivalent (2 sub-segments = 1 segment) which correspond to the mean percent volume of a segment in a 20-segment model (100 divided by 20). This approach, which does not take into account differences in segments size, has the advantage to be easier and faster to use in routine. However, to the best of our knowledge, how the SVS reliably estimates the perfusion score has never been evaluated.

In this study, we aimed to compare PVOI quantification on planar ventilation/perfusion (V/Q) lung scintigraphy assessed by a segmental visual scoring (SVS) to the Meyer score.

## Materials and methods

### Population

We reviewed the same 226 planar V/Q scans from a previous study that assessed the accuracy of various interpretation criteria of planar V/Q scintigraphy for the screening of CTEPH (13). The eligible study population consisted of consecutive patients with newly diagnosed PH, who underwent lung V/Q scintigraphy for screening CTEPH at the Brest University Hospital, France, and who were included in a French National PH registry (authorization number 842063). All patients provided written informed consent. The design and main results of the study have been previously described (13).

### Ventilation/perfusion scans acquisition and interpretation

Planar V/Q lung scans were performed according to the Société Française de Médecine Nucléaire guidelines on lung scintigraphy protocol (14). Images were acquired on a dual-head gamma cameras equipped with low energy, high-resolution, parallel-hole collimators (Intevo 16, Siemens or Symbia T6, Siemens or Ecam, Siemens). Perfusion images were obtained after intravenous administration of 140 MBq of 99mTc-macroaggregated albumin. Ventilation images were acquired after inhalation of either 99mTc-Technegas (250–700 MBq of Tc99m eluate added to the carbon crucible) or 81mKr-Krypton gas. When using Technegas, imaging started with the ventilation scan, immediately followed by the perfusion scan. When using Krypton gas, both images were acquired simultaneously. Image acquisition was performed in six views (anterior, posterior, left and right lateral, left and right posterior oblique).

A retrospective central review of all planar V/Q lung scintigraphy was performed by three nuclear physicians with different level of expertise, blinded to clinical results and to final diagnosis. Interpretation was determined via consensus reading.

Initially, the number, extent (sub segmental or segmental) and type (matched or mismatched with ventilation images) of perfusion defects were reported for each planar V/Q lung scintigraphy. A defect was visually defined as segmental if it involved more than 75% of a segment and sub-segmental if it involved less than 75% (15, 16). Small size perfusion defects involving less than 25% of a segment were not taken into account. Based on this segmental interpretation, the PVOI was calculated by multiplying the sum of defects or equivalent (2 sub-segments = 1 segment) by 5%., with the approximation that one segment represents about 5% of the total lung volume in a 20-segment model.

For the purpose of the present study, the same three nuclear medicine physicians reviewed all scans and calculated the PVOI based on the Meyer score (11). This interpretation was performed 6 months later and in a different order to avoid recall bias. According to the Meyer score, each lobe was assigned a weight based on the regional distribution of pulmonary blood flow in the supine position: right lower lobe 25%, right middle lobe 12%, right upper lobe 18%, left lower lobe 20%, lingula 12%, and left upper lobe 13%. Perfusion was then estimated, within each lobe, from the anterior, posterior and oblique views. For each lobe, a semi-quantitative perfusion score ranging from 0 to 1 (0, 0.25, 0.5, 0.75, or 1) was estimated from the film density by comparison with the photo density of an apparently normally perfused area. Each lobar perfusion score was then calculated by multiplying the corresponding lobe’s weight by the perfusion score. The overall perfusion score was determined by summing the six separate lobar perfusion scores. The percentage of vascular obstruction by perfusion scanning was then calculated as (1- Overall perfusion score) × 100. This was performed for mismatched perfusion defects and for perfusion defects regardless of the ventilation.

### Data analysis

For each planar V/Q lung scintigraphy, PVOI assessed by summing the number of segmental perfusion defects or equivalent (2 sub-segments = 1 segment = 5%) was computed on first reading. Then, PVOI assessed by Meyer method was computed on second reading 6 months later.

Because of a non-normal distribution of values, a Spearman rank correlation coefficient was calculated to evaluate correlation between the two measurement methods and plot graphs were drawn. An intra-class correlation (ICC) was calculated to assess agreement of the two methods based on a two way random effect model. A Bland et Altman plot analysis was used to evaluate agreement between the two measurements across the range of possible values. The mean difference (μ) and the standard-deviation (σ) of the differences between the two measurements were plotted against their average, and limits of agreements were defined as μ ± 1.96 * σ for both matched and mismatched evaluations, as recommended by B&A (17).

All these statistical tests were performed for mismatched perfusion defects, and for perfusion defects regardless of the ventilation (i.e., mismatched or matched defects).

Statistical analysis was done using R version 4.0– © 2009–2021 RStudio, Inc.

## Results

### Population

A total of 226 patients with newly diagnosed PH, who underwent V/Q planar scintigraphy for screening of CTEPH at the Brest University Hospital between January 2004 and January 2019 were analyzed. Out of them, 56 (25%) were eventually diagnosed with CTEPH. Among the 56 patients, 29 patients (39%) had no PE history. Among 170 patients (75%) diagnosed with non-CTEPH, 92 were classified in group 1 of PH classification (41%), 24 in group 2 (10%), 40 in group 3 (18%), 4 in group 5 (2%), and 10 were classified as having mixed causes PH (mix from group 1, 2, and 3) (4%).

### Correlation and agreement between the two measurements methods: Segmental visual scoring and Meyer score

**FIGURE 1:**
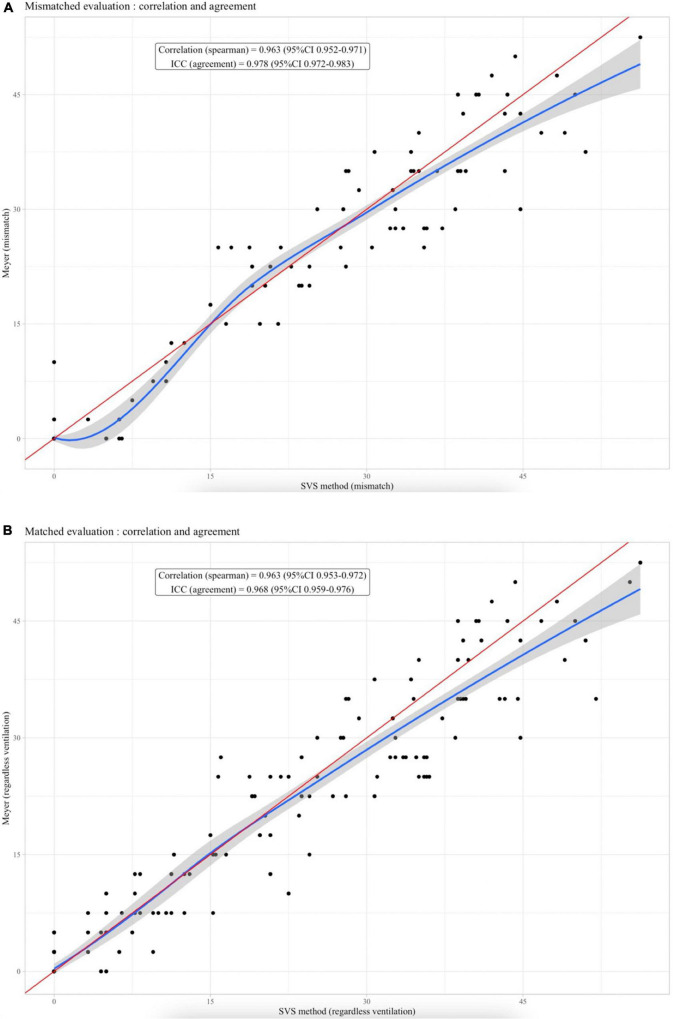
**(A)** Plot graph for correlation between SVS method and Meyer score for mismatched perfusion defect. Red line as the line of equality (*r* = 1). Blue line as local polynomial regression fitting. **(B)** Plot graph for correlation between SVS method and Meyer score for perfusion defects regardless ventilation. Red line as the line of equality (*r* = 1). Blue line as local polynomial regression fitting.

Spearman rank correlation coefficient between SVS and Meyer was 0.963 (95%CI 0.952–0.971) for mismatched perfusion defects and 0.963 (95%CI 0.953–0.972) for perfusion defects regardless of the ventilation. Intra-class correlation (ICC) for agreement was 0.978 (95%CI 0.972–0.983) for mismatched perfusion defect and 0.968 (95%CI 0.959–0.976) for perfusion defects regardless of the ventilation. Plot graph (Figures 1A,B) illustrates correlation with red line the line of equality (*r* = 1) and blue line as local polynomial regression fitting of SVS method on Meyer score, for mismatched perfusion defects or perfusion defects regardless of ventilation, respectively.

**FIGURE 2:**
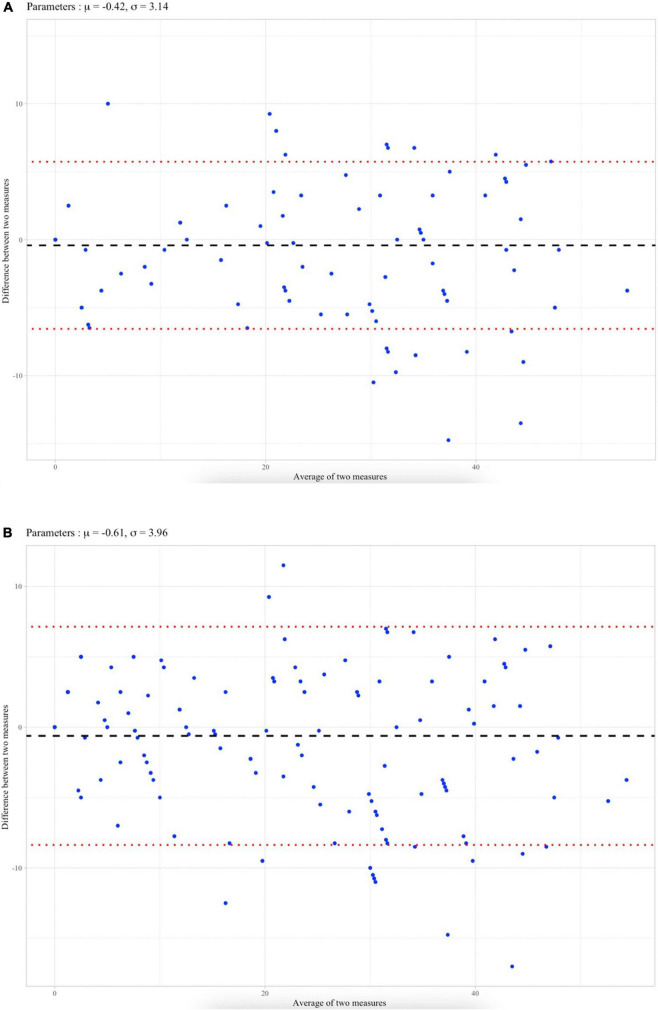
**(A)** Bland et Altman plot analysis for mismatched perfusion defect with mean difference (μ) and standard-deviation (σ). Limits of agreements were defined as μ ± 1.96 *. **(B)** Bland et Altman plot analysis for perfusion defects regardless ventilation with mean difference (μ) and standard-deviation (σ). Limits of agreements were defined as μ ± 1.96 * σ.

Concerning the Bland & Altmann analysis (Figures 2A,B), the mean difference between the SVS method and the Meyer score was 0.42 and 0.61 for the mismatched or matched evaluation, respectively. The agreement interval contained 208/226 (92%) and 212/226 (94%) of the measures for the mismatched or matched evaluation, respectively.

## Discussion

In this series of 226 patients with PH who underwent planar scintigraphy for CTEPH screening, a high correlation was found between the SVS method and the Meyer score for the quantification of the PVOI, with a correlation coefficient of 0.963 (95%CI 0.952–0.971). This result supports the use of the SVS method to quantify the PVOI in daily clinical practice.

When quantifying the PVOI based on mismatched perfusion defect, correlation and agreement between SVS and Meyer scores were excellent with a Spearman rank correlation coefficient of 0.963 (95%CI 0.952–0.971) and an ICC agreement of 0.978 (95%CI 0.972–0.983). On Bland & Altman graph (Figure 2A), the mean difference μ between SNS and Meyer scores was very close to 0.42% and the standard-deviation σ was low (3.14%). It suggests that both methods evaluate the same vascular obstruction with a standard deviation of 3.14% representing less than a segment (5%) which is unlikely to be clinically relevant.

When quantifying the PVOI based on perfusion only images (regardless of ventilation), correlation and agreement coefficients were still good with a Spearman rank correlation of 0.963 (95%CI 0.953–0.972) and an ICC agreement of 0.968 (95%CI 0.959–0.976). However, the limits of agreement between the SVS method and the Meyer score for perfusion defects regardless ventilation was wider on Bland et Altman (Figure 2B). In fact, V/Q lung scintigraphy specificity decreases if perfusion defects are interpreted regardless of ventilation images (13).

For both mismatched and perfusion only approaches, plot graphs (Figures 1A,B), which illustrate correlation between the two methods, showed that the agreement between the SVS method and the Meyer score decreased when the PVOI index was high (>30%). The Bland & Altman analysis confirmed these finding since most of the dots outside of the limits of agreements concerned a high average of two measures, which means high vascular obstruction (17). This suggests that, even if both methods seem overall very comparable to assess vascular obstruction, the difference between SVS and Meyer increases when obstruction is high. Thus, among 18 patients out of agreement limit, 14 had a PVOI over 30%. However, such errors in the quantification of very high PVOI are probably not a major issue in a clinical perspective. Indeed, for CTEPH screening, a 10% cut-off is commonly used (18). Similarly, studies reporting that the RPVO after 3–6 months of anticoagulation was an independent risk factor for PE recurrence used a diagnostic cut-off of 1 or 2 segmental perfusion defects (6). In contrast, no major discrepancy was observed in patients with a low PVOI.

**FIGURE 3:**
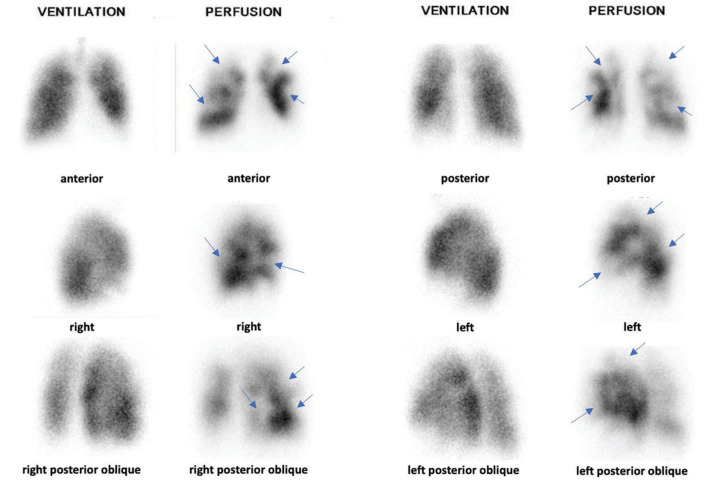
Planar V/Q lung scintigraphy with multiple mismatched perfusion defects (blue arrows). Based on Meyer score, PVOI was estimated at 50%. Right Upper Lobe (RUL): 0.5 × 18% = 9%. Middle Lobe (ML): 0.5 × 12% = 6%. Right Lower Lobe (RLL): 0.5 × 25% = 12.5%. Left Upper Lobe (LUL): 0.5 × 13% = 6.5%. Lingula: 0.5 × 12% = 6%. Left Lower Lobe (LLL): 0.5 × 20% = 10%. PVOI = 100 – (RUL + ML + RLL + LUL + LLL + Lingula) = 50%. Based on SVS method, PVOI was estimated at 45%: 9 sub-segmental mismatched perfusion defects: 9 × 5 = 45%.

Although the Meyer score was validated using pulmonary angiography as a reference standard (11) and was used in most of studies that assessed the diagnostic or prognostic value of the PVOI on V/Q scintigraphy (6, 8, 9), this score is rarely if ever used in daily practice by nuclear medicine physicians. This can be explained by the difficulty of applying the score. First, this method is based on a lobar quantification of lung perfusion, which might be complex because the exact location and delineation of each lobe is difficult to determine on planar lung V/Q scintigraphy. Second, a semi-quantitative perfusion score from 0 to 1 (0, 0.25, 0.5, 0.75, or 1) has to be assigned for each lobe based on the size and severity of the perfusion defect (Figure 3). A clear definition of how to quote this perfusion score is not provided, which may lead to interobserver variability. Third, it requires a calculation table which might be time consuming for nuclear medicine physicians in routine.

In contrast, the segmental visual scoring (SVS) has the advantage to follow the principles of interpretation of the V/Q scan for PE or CTEPH, based on a segmental approach of perfusion defects. Indeed, the PIOPED and EANM criteria used for PE diagnosis are based on the number of sub-segmental and segmental defects (2, 15, 16). After identifying the number of segmental perfusion defects (or equivalent: 1 segment = 2 sub-segments), it is easy and quick to sum by adding 5% for each segmental perfusion defects or equivalent (2 sub-segments = 1 segment). On the other hand, this multiplication factor of 5% correspond to the approximation that each segments have the same volume in a 20- segment model. It would be more accurate to use an 18-segment model as it is not possible to evaluate both lower lobe medial segments on planar lung V/Q scintigraphy. A segment would then represent 1/18 = 5.6% of the whole lungs. However, the difference between 5 and 5.6% is small and a multiplying factor of 5%, if less accurate, is much more convenient and easier to use in daily practice. Furthermore, we did not find a systematic negative bias in PVOI estimation as the mean difference of PVOI calculated with the SMS and the Meyer score was 0.42 and 0.61 for the mismatched or matched evaluation, respectively. Finally, there are two regions of the left lung in which two segments are joined as one as they have a common tertiary segmental bronchus: the left upper lobe apico-posterior segment and the left lower lobe antero-medial segment. However, this anatomical segmentation does not make sense for PVOI quantification as it is not correlated with the segments size.

What are the clinical implications of our findings? PVOI quantification has been described to be of particular relevance in various clinical scenarios. In patients with PE, PVOI at diagnosis or after a minimum of 3 months of anticoagulant therapy has been described as an independent risk factor of PE recurrence, and may be used to adapt the duration of anticoagulant therapy (6, 8, 9). In patients with PH assessed for screening CTEPH, PVOI quantification may allow for a better selection of patients for whom CTEPH could be confidently excluded from those in whom additional testing in an expert center is required. PVOI quantification may also be of value for therapeutic assessment and follow up of patients with CTEPH, especially in patients treated with pulmonary balloon angioplasty (10, 19, 20). Because of the complexity of the Meyer score, the PVOI was rarely reported in daily practice. Our study shows that the SVS method, in addition to be easy to learn and apply, is a reliable method for PVOI quantification.

Our study has some limitations. First, we performed a consensus reading and did not assess interobserver reproducibility. However, principles of interpretation of the SVS method, based on the recognition of mismatched perfusion defects are well-known by nuclear medicine physicians. In contrast, the Meyer score with its lobar approach is unusual, and Wan et al. (12), reported an interobserver agreement of only 0.71 in a series of 65 patients. Second, all lung V/Q scans were performed for screening CTEPH. It would be of interest to confirm these findings in patients with acute PE, both at diagnosis and after completion of anticoagulant therapy. On the other hand, the principle of interpretation based on the recognition of perfusion segmental defects is similar for both conditions. Third, we only focused the analysis on planar lung V/Q scintigraphy. The advent of SPECT/CT imaging offers a great opportunity to improve the accuracy of PVOI quantification (21, 22), although there is not a reference method for comparison as the Meyer score has not been validated on SPECT imaging.

## Conclusion

Our study shows a high correlation and low differences in PVOI quantification on V/Q planar scintigraphy when using a segmental visual scoring (SVS) as compared to the Meyer score. The SVS has the great advantage to be easy to learn and apply in daily practice. As PVOI quantification may have a prognostic implication in thrombo-embolic diseases, it could be systematically calculated using the SVS and provided in lung scan reports.

## Data availability statement

The raw data supporting the conclusions of this article will be made available by the authors, without undue reservation.

## Ethics statement

The studies involving human participants were reviewed and approved by the French National PH Registry (authorization number: 842063). The patients/participants provided their written informed consent to participate in this study.

## Author contributions

RLP, CT, PL, PS, and CO performed the material preparation, data collection, and analysis. RLP and PL wrote the first draft of the manuscript. All authors contributed to the study conception and design, commented on previous versions of the manuscript, and read and approved the final manuscript.
